# Account of the Efficacy of Sugar of Lead, in a Case of Epilepsy. In a Letter to Dr. R. Coxe, of Philadelphia

**Published:** 1807-10

**Authors:** T. R. P. Spence

**Affiliations:** Accomac County, Virginia


### *Account of the Efficacy of Sugar of Lead, in a Case of Epilepsy. In a Letter to Dr. R. Coxe, of Philadelphia. By* Dr. T. R. P. Spence, *of Accomac County, Virginia.*

Sir,

The duty I owe society, the medical faculty in general, and Drs. Church, Wistar, and Rush in particular, induces me to make this communication, being a history of my own case of epilepsy—requesting for it, if you see proper, a place in your Museum.

My first attack was in the 23d year of my age. On October 13th, 1803, as I was riding, I fell from my horse, without any premonitory symptom whatever, and remained insensible (by estimation of the person with me) half an hour. The fit was succeeded by a violent pain in my head, a full, slow, and hard pulse, not exceeding sixty-four strokes in a minute. Being confident it was an epileptic attack, from being hereditarily predisposed,* I immediately lost sixteen ounces of blood, and pursued an antiphlogistic course, tolerably strict, until my system was reduced

---

* My elder brother was first attacked at about the same age; another had it from his infancy, and died about the age of puberty.

reduced pretty considerably; I then begun with the cuprum ammoniacum, and continued some time; but was subject to attacks, or fulness and pain in my head, at every full and change of the moon; which leads me to believe the human system, particularly in this disease, is subject to lunar influence; for when I did not recollect the full and change, I could discover it by my feelings, particularly by a sensation of fulness and pain in my head.

I continued more or less subject to attacks of the same kind until 27th of May, 1804: I went to Philadelphia, and consulted the above mentioned medical gentlemen, who recommended me to persevere in the plan I had adopted, and to make trial of mercury, zinc, and lead. I first began with mercury, and found that it would suspend the disease, particularly the pain in the head, for two months; but when the system was unimpregnated with mercury, would immediately have an attack, at the next lunar period; which induced me to keep up for seven months, a pretty considerable ptyalism, and twice a salivation. I must also observe, that I used bleeding once a month, regularly. My pulse during this time was from 78 to 90, but seldom exceeded 84 in a minute.

I experienced no inconveniency from the mercury, more than the sore mouth. There was a greater discharge of urine during its use; and that quite transparent, which had not been the case for three years previously.

Having considerable practice during the autumnal months I found that riding on horseback in the sun, at this season, produced great fatigue, and operated as an exciting cause, affecting my head very much with pain. I had only four fits from the 27th May until the 6th December of the same year. All kinds of exercise producing fatigue had similar effects; but I think shooting was most injurious. The report of a gun would affect my head and pulse. I was pleased with the amusement, and undertook it on horseback to prevent the fatigue of walking; but the report was still injurious. I was most subject to the complaint after going to bed, during the inaction of sleep. In September 1804, I was so much reduced by the regimen and medicine, that I left off the mercury during this month, and tried the cold and shower bath; but with considerable disadvantage. Two attacks during this month. I then continued the medicine, October and November, and had another fit after gunning, on the 6th of December.

All hopes of recovery being nearly extinguished, and life life irksome, I determined to give the sugar of lead as fair a trial as I had done the mercury, and to push it to a considerable extent, let the consequence be what it might. The good effects of its use in children by Dr. Rush, gave some encouragement; while the insignificancy attending its use in adults, mingled rather more fears than hopes. In several cases in which the Doctor used it, not one case was cured; and only one was suspended a few weeks. I therefore, with reluctance, began and took of sacch. saturni one-fourth of a grain three times a day, and increased the dose in one week to one grain; and continued with that for the space of two weeks, and then increased it to eight grains twice a day; and continued taking it in this quantity three or four weeks, with, I may say, happy effects; not one symptom of my former complaint all this time; my pulse from 78 to 84 in a minute.

The sensible effect of the lead for the first two weeks, was only a little costiveness; afterwards, a sponginess of the gums came on, with a dark livid appearance around the dentes incisores; small flow of saliva; fœtid breath; although my teeth were loose, my mouth was not sore; a great increase of urine, more than when I took mercury; costiveness, sometimes for two days, and then the fæces quite black and compact; my upper extremities, particularly my elbows, affected with pain in the morning.

Although apprehensive of serious consequences, I still determined to go on with the medicine, and lose some blood to assist it; accordingly, on the evening of the 24th January I lost sixteen ounces; my arm bleeding accidentally the next morning, I lost, by estimation, eleven ounces more. I now, for the first time, lost all appetite for food, and continued three or four days without eating any thing; this was succeeded by pain in the epigastrium, extending down below the navel, in the spine, and region of the liver, which continued ten days, very excruciating indeed; I took laxatives, opiates, in large and repeated doses, together with a long catalogue of medicines. without relief for the time above specified.

The pains continued very obstinate, but less severe, about six weeks; those in my arms were not gone, and extending to my legs, rendered standing impracticable. During the continuace of these pains I was bled five times; the blood after standing twelve hours had very little or no serum; and the crassamentum was of a dark livid appearance.

After trying almost every medicine that imagination could could suggest, and being in a state of slow convalescency I found the greatest effectual relief from chalybs ppt. and myrrh; this first restored my appetite.* Since recovering my health, my stomach appears to be so much contracted, that I cannot eat more than two-thirds or half as much as I could, or was wont to eat before, without a sensation of considerable fulness. During the artificial disease from lead, my pulse did not exceed 60 or 64 strokes in a minute; and since, from 78 to 84 is its natural standard.

It has now been seven months since I began with the internal use of the sugar of lead; since which time I have not had the least sign of my former epileptic complaint; except once, after fatigue in the hot sun, I had considerable pain in my head.

I was bled every month during the time I used mercury, without any sensible relief from it; and am led to believe it an injurious plan to pursue in this complaint, from the following consideration. Hemorrhage, or blood-letting to excess, will produce plethora, and I believe where that is used in a healthy state of the system, as in epilepsy where there is no *general* morbid excitement, it must do harm by occasioning a predisposition. Moreover, if it has any good effect, that good effect is lost by not attending particularly to the operation at a particular period of the moon; which, when neglected, renders the attacks more frequent and more violent.

*Queries.*—Whether or not has my taking the sugar of lead so soon after a course of mercury, produced those happy effects of relief, which were not obtained in adults by the use of the same medicine?

Whether or not have the good effects of the medicine proceeded from its direct sedative operation; or from its contracting the stomach, and by that means obviating plethora?

What

* I was very sanguine in my expectations from opium, on hearing my friend Dr. I. Stevenson say, "That he attended the case of two children, one seven, the other nine years old, who eat a quantity of white lead for chalk: they both had very severe pains in the abdomen, occurring at intervals; their skins were remarkably yellow; their bowels were nearly natural or rather costive; laxatives produced very black stools, but no alleviation of pain; opium was so good a palliative that he had recourse to nothing else; it seemed plainly indicated, neither of their pulses beat oftener than 36, and very soft; opium rendered the intervals of ease longer and longer, until the pains vanished altogether in about five or six days."

What is the *modus operandi* of saccharum saturni taken internally? Is it a sedative or a stimulant?

Perhaps the pen of some speculative theorist might be advantageously employed in answering these queries. I am content with having experienced the beneficial effects of the medicine, and giving a narrative of facts and circumstances which led me to it.

These facts I have thrown together without much regard to order, or systematic arrangement; they are related with all the fidelity my memory will admit of; I wish I could have avoided reasoning on them? but as a celebrated author observes—" As thorny as the subject appears we must approach and handle it;" perhaps they may serve as one round in the ladder, by which we may finally climb to the summit of medical science.

*July* 28, 1805.

